# Power and sample sizes estimation in clinical trials with treatment switching in intention-to-treat analysis: a simulation study

**DOI:** 10.1186/s12874-023-01864-1

**Published:** 2023-02-23

**Authors:** Lejun Deng, Chih-Yuan Hsu, Yu Shyr

**Affiliations:** 1 Montgomery Bell Academy, Nashville, TN 37205 USA; 2 Department of Biostatistics, Vanderbilt University Medical Center, Nashville, TN 37232 USA; 3 Center for Quantitative Sciences, Vanderbilt University Medical Center, Nashville, TN 37232 USA

**Keywords:** Crossover, Intention-to-treat analysis, Randomized controlled trials, Treatment switching

## Abstract

**Background:**

Treatment switching, also called crossover, is common in clinical trials because of ethical concerns or other reasons. When it occurs and the primary objective is to identify treatment effects, the most widely used intention-to-treat analysis may lead to underpowered trials. Here, we presented an approach to preview power reductions and to estimate sample sizes required to achieve the desired power when treatment switching occurs in the intention-to-treat analysis.

**Methods:**

We proposed a simulation-based approach and developed an R package to perform power and sample sizes estimation in clinical trials with treatment switching.

**Results:**

We simulated a number of randomized trials incorporating treatment switching and investigated the impact of the relative effectiveness of the experimental treatment to the control, the switching probability, the switching time, and the deviation between the assumed and the real distributions for the survival time on power reductions and sample sizes estimation. The switching probability and the switching time are key determinants for significant power decreasing and thus sample sizes surging to maintain the desired power. The sample sizes required in randomized trials absence of treatment switching vary from around four-fifths to one-seventh of the sample sizes required in randomized trials allowing treatment switching as the switching probability increases. The power reductions and sample sizes increase with the decrease of switching time.

**Conclusions:**

The simulation-based approach not only provides a preview for power declining but also calculates the required sample size to achieve an expected power in the intention-to-treat analysis when treatment switching occurs. It will provide researchers and clinicians with useful information before randomized controlled trials are conducted.

**Supplementary Information:**

The online version contains supplementary material available at 10.1186/s12874-023-01864-1.

## Background

Clinical trials are often used to assess the effectiveness of new treatments. The most reliable clinical trial is the randomized controlled trials (RCT). In an RCT, patients are randomly assigned to the control group, where they receive a placebo or an existing standard treatment, or the experimental group, where they receive the experimental treatment. For treatments against diseases like cancer, the effectiveness of the new treatment is determined by comparing the observed survival time of patients in the experimental group to those in the control group. The power of a statistical test, which is the probability to correctly tell if the experimental treatment is better when it is truly better, is extremely important. Being able to correctly determine the effectiveness of an experimental treatment gives justification for its production, allows for better utilization of resources, and helps those who will benefit from the experimental treatment.

A common method used to interpret the data from a RCT is the intention-to-treat analysis (ITT). The ITT analysis includes all patients with randomization in statistical analysis and compares their responses to determine the effectiveness of the experimental treatment according to the treatment group that was initially assigned to them, regardless of what treatment they actually received. Patients, however, do not always comply with the treatment they were assigned to. Because of ethical concerns or other reasons, they may switch treatments from the control group to the experimental group. For example, it may occur when a disease progresses or when the healthcare provider believes the patient’s prognosis will improve with the experimental treatment. When treatment switching, also called crossover, is permitted, the ITT analysis is confounded, which decreases the power of a statistical test. A simple and alternative approach, such as the per-protocol analysis, excludes the participants who switch treatments from the statistical analysis. Nevertheless, this may heavily bias the analysis results if the participants included and excluded in the analysis significantly differ in prognosis, i.e., treatment switching is associated with prognostic variables [1].

Various statistical methods dealing with treatment switching have been proposed. Law and Kaldor [2] proposed the adjusted Cox model by splitting the study population into four groups according to their initial and final treatment groups and by assuming different hazard functions with time dependent covariates defined by the time of switching treatment for the four group. Loeys and Goetghebeur [3] proposed the causal proportional hazards estimator by assuming that patients in one arm complete their treatment while the patients in the other arm either completely fulfills their treatment or does not fulfill their treatment at all. Robins and Tsiatis [4] proposed correcting for non-compliance by using the rank preserving structural accelerated failure time models (RPSFT). Based on the RPSFT, Branson and Whitehead [5] and Zhang and Chen [6] provided iterative parametric estimation and modified iterative parametric estimation methods, respectively, for fast and reliably estimating the treatment effect. Morden et al. [1] and Latimer et al. [7, 8] performed simulations studies to compare several adjustment methods mentioned above.

However, sample sizes estimation in most RCTs is still based on the assumption of no treatment switching and predetermined statistical tests, such as the logrank test in ITT analysis. Given the sample sizes estimated from no-switching designs, the ITT analysis may underestimate the positive treatment effect when treatment switching occurs. In this study, we proposed a simulation-based approach, *PowerSwitchingTrial*, to preview power reductions and calculate sample sizes required in RCTs allowing treatment switching. We investigated the impact of the relative effectiveness of the experimental treatment to the control, the switching probability, the switching time, and the deviation of the assumed survival distribution from the real one on power reductions and sample sizes estimation. We found the switching probability and the switching time are key determinants for power decreasing and thus sample sizes increasing to maintain the desired power in ITT analysis. *PowerSwitchingTrial* is freely available at https://github.com/darwin-hub/PowerSwitchingTrial.

## Methods

### *PowerSwtichingTrial* overview

**Fig. 1:**
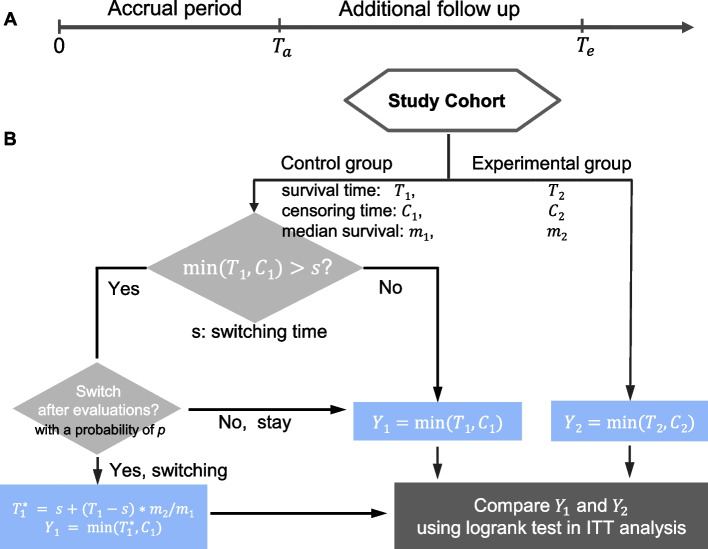
A flowchart of *PowerSwtichingTrial*

*PowerSwitchingTrial* is a simulation-based R package, developed for exploring statistical powers and sample sizes required in RCT trials allowing treatment switching when the logrank test is used in ITT analysis. The flowchart of *PowerSwitchingTrial* is shown in Fig. 1.

*PowerSwtichingTrial* simulates a two-arm randomized clinical trial study with an accrual time of *T*_*a*_ and an additional follow-up time of *T*_*e*_ − *T*_*a*_, where *T*_*e*_ is the time from the start to the end of the trial. Participants are assumed to enter the study uniformly during the accrual time period, i.e., the entry time follows a uniform distribution *U*(0, *T*_*a*_), and soon be randomly assigned to one of the two treatment groups, the control group or the experimental group, with an allocation ratio of *r* (*r* is the ratio of the numbers of participants in the experimental and the control groups). *T*_*a*_ = 0 indicates all participants enter the study at the beginning of the study. The survival time of participants in the control and the experimental groups, denoted by *T*_1_ and *T*_2_, is assumed to follow exponential distributions with median values of *m*_1_ and *m*_2_, respectively. The censoring time of participants in the two groups, denoted by *C*_1_ and *C*_2_, is defined as the time from randomization to dropout or to the end of the trial if participants do not experience the event of interest. The censoring consists of both dropout censoring and administrative censoring. The distributions of the censoring time for *C*_1_ and *C*_2_ are assumed to be the same and can be formulated as follows [9]:

$$f\left(c|v\right)=d(c)I\left(0<c<{T}_e-v\right)+\overline{D}\left({T}_e-v\right)I\left(c={T}_e-v\right),$$

where *v* is the entry time, and *d*(*c*) and $\overline{D}(c)$ are the density function and survival function of the dropout censoring, respectively. *I*(∙) is the indicator function. For simplicity, we assume a uniform distribution for the dropout censoring, i.e., *d*(*c*) = *h*^−1^ *I*(0 < *c* < *h*), and *h* is determined by the formula of *P*(*C*_1_ < *T*_1_) with a given censoring rate of the control group under no treatment switching. *P*(*C*_1_ < *T*_1_) is a function of *h* and can be explicitly expressed (See Supplementary Materials for details).

*PowerSwtichingTrial* allows participants in the control group have a chance to switch to the experimental group if predetermined conditions are satisfied. For example, if patients with cancer have a disease progression before death (assume death is the event of interest), they may switch from the standard treatment to the new treatment after disease progression and being evaluated by the investigator [10]. The switching time, denoted by *s*, is the time from randomization to the moment when a participant may switch with a probability *p*. *p*, defined as the switching probability*,* is the probability that a participant who qualifies for treatment switching will switch from the control group to the experimental group after being evaluated by physicians or health care professionals. Five options were provided for the switching time. The first three options assume *s* is correlated with *T*_1_ and *s* = *X* × *T*_1_, where *X* is independent of the survival time *T*_1_ and follows one of the three distributions, beta, gamma, or uniform *U*(0, 1). Since most often patients are allowed to switch to the new therapy at the point of disease progression, the assumption that switching time is correlated with the survival time is appropriate. The factor *X* controls the distribution of the switching time and the correlation between the switching time and the survival time *T*_1_ by the three different distributions and the associated parameters. If *X* follows beta or uniform distributions, *s* < *T*_1_, denoting the switching time is always less than the survival time. In contrast, if *X* follows gamma distributions, *s* has a chance to be greater than *T*_1_. The parameters in the beta and the gamma distributions are determined by given parameters *pt* and *rho* (Supplementary Material). *pt* = *E*(*s*)/*E*(*T*_1_) denotes the ratio of the average switching time to the average survival time of the control group and *rho* denotes the correlation between *s* and *T*_1_. If the switching time is the time when disease progresses, *pt* and *rho* can be estimated by the time of progression and the time of events from previous studies. If *X* ~ *U*(0, 1), then *pt* = 0.5 and *rho* is a constant of 0.775 when *T*_1_ follows an exponential distribution (Supplementary Material). The other two options assume that *s* is independent of *T*_1_, where *s* either follows an exponential distribution with its mean determined by a given *pt*, or *s* is a constant. The survival time for participants starting from switching is assumed to increase by a constant multiplier of *A*, based on the rank preserving structural failure time model [4]. That is, the survival time of the participants with treatment switching will be ${T}_1^{\ast }=s+\left({T}_1-s\right)\times A$, *A *= *m*_2_/*m*_1_. Therefore, the observable survival time *Y*_1_ for the participants who are originally assigned to the control group will be equal to *min*(*T*_1_, *C*_1_) without treatment switching and $\mathit{\min}\left({T}_1^{\ast },{C}_1\right)$ with treatment switching. The observable survival time *Y*_2_ for the participants in the experimental group will be equal to *min* (*T*_2_, *C*_2_). Finally, the logrank test is used to compare *Y*_1_ and *Y*_2_.

### Two main functions of *PowerSwtichingTrial*

*PowerSwtichingTrial* provides two main functions, *LogRankTestMix2PowerMedian* and *LogRankTestMix2Nmedian****. ****LogRankTestMix2PowerMedian* calculates the power, given sample sizes, the difference in the survival time between two groups, and the other parameters. *LogRankTestMix2NMedian* estimates sample sizes, given an expected power to detect the difference in the survival time between two groups, and the other required parameters.

*LogRankTestMix2PowerMedian*, a function to calculate the power, includes 13 input parameters to simulate a number of scenarios, *n*, *m*_1_, *m*_2_, *r*, *T*_*a*_, *T*_*e*_, *p*, *alpha*, *s.dist*, *pt*, *rho*, *censor.rate* and *reps*. *n* is the number of participants in the control group, *m*_1_, *m*_2_, *r*, *T*_*a*_, *T*_*e*_, and the switching probability (*p*) were defined in the Section 2.1, and *alpha* is the significance level. *s.dist* is the option for the switching time, which should be one of the five options: “beta”, “gamma”, “unif”, “indepExp”, or a constant value. Those parameters in the beta, gamma, or independent exponential distributions (“indepExp”) are determined by the given *pt* and *rho* (Section 2.1 and Supplementary Material). *censor.rate* is a predetermined censoring rate of the control group under no treatment switching. When *censor.rate* = “AC.only”, it assumes no dropout censoring but administrative censoring only. *Reps* is the number of computer simulations. The function will return the power, that is, the average number of rejecting the null hypothesis which suggests no difference in survival time between the two treatment groups across *reps* simulations, and the expected number of events for the two groups.

*LogRankTestMix2Nmedian is* a function to calculate the required sample sizes to meet a given power and other settings. *LogRankTestMix2NMedian* uses the same parameters as *LogRankTestMix2PowerMedian*, except the parameter of *n* being replaced by *power*, *lower*, and *upper*. The lower and upper is the minimum and maximum sample sizes that users set to explore. The function uses the bisection method to find the sample size (*n*) required between the *lower* and *upper* bounds.

## Results

### A scenario from a real clinical trial

We first demonstrated the application of the two R functions using a real clinical trial setting. The study was an open-label phase III trial to compare the benefit of survivals in patients with chemotherapy-refractory metastatic colorectal cancer who were randomly assigned to either panitumumab + best supportive care (BSC) or BSC alone [10]. After 1040 patients were screened, 231 patients were randomly assigned to panitumumab + BSC and 232 to BSC alone. The maximum follow-up time was 113 weeks (~ 26 months). Thus, we assumed *T*_*a*_ = 0 and *T*_*e*_ = 26. Among BSC alone patients, 85% had disease progression, and 76% crossed over to panitumumab + BSC after being evaluated by the investigator. That is, the switch probability *p* was 0.89 (=0.76/0.85). According to their ITT results reported on ClinicalTrial.gov (NCT00113763), the median of overall survival (OS) in the panitumumab + BSC group was 6.4 months (*m*_2_ = 6.4) and the median of OS in the BSC alone group was 6.2 months (*m*_1_ = 6.2). There was no significant difference between the two treatment groups.

When there was no treatment switching, we assumed the actual OS median in the BSC alone would be shorter than 6.2 months since the panitumumab + BSC group had better progression-free survival than the BSC alone (logrank test, *p*-value < 0.0001). If the actual OS median in the BSC alone was 4.43 months (*m*_1_ = 4.43), the power to detect the difference between two treatment groups could attain 0.9 at the significance level of 0.01 with the sample size *n*= 232 and *r* = 1.

When the treatment switching was allowed, with the sample size *n*= 232 and *r* = 1, the power to detect the difference in OS were around 0.02–0.10 and 0.07 assuming *s.dist* = “gamma” and “indepExp”, respectively. That is, it was hard to detect the difference in OS between two treatment groups, which was consistent with the original result. To achieve a power of 0.9 at the significance level of 0.01, the sample size needs to be more than 2000 (Table 1). Here, we chose *s.dist* = “gamma” (i.e., *s* = *XT*_1_ and *X* follows a gamma distribution) since some patients didn’t experience disease progression prior to death. The parameters of shape and rate parameters in the gamma distribution were determined by *pt* and *rho*, where *pt* = 0.3 (=1.96/6.4). *pt* was estimated by the ratio of *E*(*s*) = 1.96 months (from the reported mean PFS time, 8.5 weeks) and *E*(*T*_1_) = 6.4 months (= *m*_1_ /log (2)). We considered *rho*= 0.1, 0.3, 0.5, 0.7, and 0.9 to model low to high correlations between PFS and OS since *rho* was unknown. In addition, we chose *s.dist* = “indepExp” (i.e., *s* is independent of *T*_1_ and follows an exponential distribution), where *E*(*s*) = 0.3*E*(*T*_1_). The censoring rate was set to be 0.02 (=4/232) since 4 censoring cases were reported in the 232 BSC alone patients.

**Table 1:** Powers and required sample sizes in a real clinical trial

	*s.dist* = “gamma”					*s.dist* = “indepExp”
*rho* (correlation of ***s*** with ***T***_**1**_)	0.1	0.3	0.5	0.7	0.9	0
Power at the significance level of 0.01 with ***n=*** 232 and ***r*** = 1	0.024	0.060	0.093	0.104	0.099	0.074
Required sample sizes (***n***) to attain the power of 0.9 at the significance level of 0.01	> 10,000	3339	2276	2034	2084	2828

### Simulation scenarios

A number of simulation scenarios were generated to explore the effect of treatment switching on powers and sample sizes estimation in ITT analysis, which included the relative different effectiveness of the experimental treatment to the control, the different switching probability, the different types of switching time, and the deviation of the assumed distribution from the real distribution. In each scenario, the accrual time *T*_*a*_ was set to 3, the study time *T*_*e*_ was set to 5, and *m*_1_ was set to 1. Under no treatment switching, the expected power to detect the survival difference between two treatment groups was set as 0.8 and the significance level was set as 0.05. We also set *r* = 1, *pt* = 0.5, *rho* = 0.775, and *censor.rate* = 0.2. The number of computer simulations was set at 5000.

#### Effect of the relative effectiveness on power and sample sizes estimation

We first investigated the impact of the relative effectiveness of the experimental treatment to the control (*m*_2_/*m*_1_) on power and sample sizes estimation, which ranged at 1.5 and 2. The power decreased slightly and the ratio (*n*_*s*_/*n*_*no* − *s*_) of sample sizes with treatment switching (*n*_*s*_) to those absence of switching (*n*_*no* − *s*_) increased with the rising relative effectiveness (Table 2). For example, when *m*_2_/*m*_1_= 1.5 and *p* = 0.4, *n*_*s*_/*n*_*no* − *s*_ = 1.65 (*n*_*no* − *s*_ = 130 and *n*_*s*_ = 215) if *s.dist* = “beta”. When the relative effectiveness grew from 1.5 to 2, the ratio *n*_*s*_/*n*_*no* − *s*_ increased from 1.65 to 1.81, and the power decreased from 0.57 to 0.56. The changes in the ratios and powers in the four types of random switching time had similar trends. The change with *s.dist* = “indepExp” was the smallest, but larger sample sizes were required.

**Table 2:** Simulation results for powers and sample sizes

***p***		no switching	switching s.dist = “beta”	switching s.dist = “gamma”	switching s.dist = “unif”	switching s.dist = “indepExp”
***m***_**2**_/***m***_**1**_ ***=*** **1.5**						
0.2	*n*	130	166	165	168	169
	E1	104.0	130.8	130.1	132.5	132.8
	E2	89.2	113.8	113.2	115.1	115.8
	Power^a^	0.80	0.70	0.70	0.70	0.69
	*n*_*s*_/*n*_*no* − *s*_	–	1.28	1.27	1.29	1.30
0.4	*n*	130	215	219	217	235
	E1	104.0	167.0	169.9	168.5	180.9
	E2	89.2	147.6	150.2	148.9	161.1
	Power^a^	0.80	0.57	0.58	0.57	0.54
	*n*_*s*_/*n*_*no* − *s*_	–	1.65	1.68	1.67	1.81
0.6	*n*	130	294	301	297	344
	E1	104.0	225.0	230.0	227.1	259.4
	E2	89.2	201.7	206.4	203.9	236.0
	Power^a^	0.80	0.47	0.45	0.48	0.40
	*n*_*s*_/*n*_*no* − *s*_	–	2.26	2.32	2.28	2.65
0.8	*n*	130	418	437	416	534
	E1	104.0	314.7	328.5	313.3	394.8
	E2	89.2	286.4	299.6	285.2	366.4
	Power^a^	0.80	0.35	0.33	0.35	0.29
	*n*_*s*_/*n*_*no* − *s*_	–	3.22	3.36	3.20	4.11
1.0	*n*	130	627	782	657	937
	E1	104.0	464.9	578.1	487.2	678.4
	E2	89.2	430.3	536.3	450.7	642.2
	Power^a^	0.80	0.24	0.23	0.24	0.18
	*n*_*s*_/*n*_*no* − *s*_	–	4.82	6.02	5.05	7.21
***m***_**2**_/***m***_**1**_ ***=*** **2**						
0.2	*n*	48	64	63	62	64
	E1	38.4	49.9	48.9	48.2	49.4
	E2	28.4	38.1	37.4	36.9	38.0
	Power^a^	0.80	0.69	0.69	0.69	0.68
	*n*_*s*_/*n*_*no* − *s*_	–	1.33	1.31	1.29	1.33
0.4	*n*	48	87	84	84	88
	E1	38.4	65.8	63.5	63.7	65.7
	E2	28.4	51.8	49.9	49.9	52.2
	Power^a^	0.80	0.56	0.57	0.56	0.53
	*n*_*s*_/*n*_*no* − *s*_	–	1.81	1.75	1.75	1.83
0.6	*n*	48	114	124	116	127
	E1	38.4	83.8	90.7	85.1	92.1
	E2	28.4	67.5	73.5	69.0	76.0
	Power^a^	0.80	0.44	0.42	0.45	0.40
	*n*_*s*_/*n*_*no* − *s*_	–	2.38	2.58	2.42	2.65
0.8	*n*	48	167	182	167	202
	E1	38.4	119.0	129.0	118.9	140.0
	E2	28.4	99.1	108.1	99.0	119.8
	Power^a^	0.80	0.32	0.31	0.31	0.27
	*n*_*s*_/*n*_*no* − *s*_	–	3.48	3.79	3.48	4.21
1.0	*n*	48	265	296	269	355
	E1	38.4	182.7	203.3	185.7	236.2
	E2	28.4	157.4	175.7	159.4	210.7
	Power^a^	0.80	0.23	0.22	0.23	0.19
	*n*_*s*_/*n*_*no* − *s*_	–	5.52	6.17	5.60	7.40

^a^Power at the sample size *n*_*no* − *s*_. E1 and E2: the expected numbers of events in the control group and the experimental group. *n*_*s*_ and *n*_*no* − *s*_ denote the required sample size with and without treatment switching, respectively at the power of 0.8 and the significance level of 0.05

#### Effect of the switching probability on power and sample sizes estimation

We further evaluated the effect the switching probability (*p*) on power and sample sizes estimation, which was set at 0.2, 0.4, 0.6, 0.8, and 1.0. The power decreased and the ratio of sample sizes (*n*_*s*_/*n*_*no* − *s*_) increased significantly with the increased switching probability (Table 2). At *m*_2_/*m*_1_= 1.5, when the switching probability increased from 0.2 to 1.0, the ratio of sample sizes *n*_*s*_/*n*_*no* − *s*_ increased from 1.28 to 4.82 if *s.dist* = “beta”, 1.27 to 6.02 if *s.dist* = “gamma”, 1.29 to 5.05 if *s.dist* = “unif”, and 1.30 to 7.21 if *s.dist* = “indepExp”. The power decreased from 0.70 to 0.24 if *s.dist* = “beta”, 0.70 to 0.23 if *s.dist* = “gamma”, 0.70 to 0.24 if *s.dist* = “unif”, and 0.69 to 0.18 if *s.dist* = “indepExp”. The changes of power and sample sizes were similar at *m*_2_/*m*_1_= 2. The results indicate that the switching probability is critical for power and sample sizes estimation. A small change of the switching probability would require much more sample sizes to maintain the expected power.

#### Effect of the switching time on power and sample sizes estimation

In addition, we explored the effect of the switching time on power and sample sizes estimation. We used the constant switching time (*s*) to make it easy to understand the impact. The switching time was set at 0.5/log (2) and 1/log (2), which were equivalent to *pt* = 0.5 and 1. The ratio of sample sizes (*n*_*s*_/*n*_*no* − *s*_) increased and power decreased significantly when the switching time advanced, especially when the switching probability *p* was greater than 0.6. For example, the ratio of sample sizes decreased from 1.91 to 4.83 when the switching time advanced from 1/log (2) to 0.5/log (2) under *p* = 1 and *m*_2_/*m*_1_ = 1.5 (Table 3). The results indicate that the switching time is also a key factor for power reductions and sample sizes increase in clinical trials with treatment switching. More simulation results with different *m*_2_/*m*_1_ and *r* were included in Supplementary Table s1 and s2. The change of the allocation ratio *r* had very small effect on sample sizes and power.

**Table 3:** Effect of the switching time on power and sample sizes

		***m***_**2**_/***m***_**1**_ ***=*** 1.5			***m***_**2**_/***m***_**1**_ ***=*** 2		
***p***		no switching	switching s = 0.5/log(2)	switching s = 1.0/log(2)	no switching	switching s = 0.5/log(2)	switching s = 1.0/log(2)
0.2	*n*	130	165	144	48	63	55
	E1	104.0	129.7	114.1	38.4	48.9	43.2
	E2	89.2	113.2	98.7	28.4	37.4	32.7
	Power^a^	0.80	0.71	0.76	0.80	0.70	0.76
	*n*_*s*_/*n*_*no* − *s*_	–	1.27	1.11	–	1.31	1.15
0.4	*n*	130	217	165	48	83	63
	E1	104.0	167.4	129.3	38.4	62.3	48.7
	E2	89.2	148.9	113.2	28.4	49.2	37.4
	Power^a^	0.80	0.59	0.71	0.80	0.57	0.70
	*n*_*s*_/*n*_*no* − *s*_	–	1.67	1.27	–	1.73	1.31
0.6	*n*	130	290	187	48	113	71
	E1	104.0	219.8	145.1	38.4	82.8	53.9
	E2	89.2	198.7	128.3	28.4	67.8	42.2
	Power^a^	0.80	0.47	0.65	0.80	0.46	0.65
	*n*_*s*_/*n*_*no* − *s*_	–	2.23	1.44	–	2.35	1.48
0.8	*n*	130	427	214	48	164	81
	E1	104.0	317.4	164.4	38.4	115.2	60.4
	E2	89.2	292.8	146.8	28.4	97.4	48.1
	Power^a^	0.80	0.36	0.59	0.80	0.35	0.59
	*n*_*s*_/*n*_*no* − *s*_	–	3.28	1.65	–	3.42	1.69
1.0	*n*	130	628	248	48	247	93
	E1	104.0	458.1	188.3	38.4	167.1	68.1
	E2	89.2	430.8	170.0	28.4	146.7	55.2
	Power^a^	0.80	0.26	0.53	0.80	0.25	0.53
	*n*_*s*_/*n*_*no* − *s*_	–	4.83	1.91	–	5.15	1.94

^a^Power at the sample size *n*_*no* − *s*_. E1 and E2: the expected numbers of events in the control group and the experimental group. *n*_*s*_ and *n*_*no* − *s*_ denote the required sample size with and without treatment switching, respectively

### Power estimation when true distributions deviate from the assumed distributions

Finally, we performed simulation studies to investigate the robustness of power if the true distributions for the survival time deviate from the assumed exponential distributions. We first calculated the required sample sizes based on the survival time following the exponential distributions, given *power* = 0.8, *m*_1_ = 1, *m*_2_ = 1.5, *r* = 1, *T*_*a*_ = 3, *T*_*e*_ = 5, *alpha* = 0.05, *pt* = 0.5, *rho* = 0.775, and *censor.rate* = 0.2. Then, we assumed the true distributions of the control and the experimental groups follow Weibull distributions with the same median survival time as those in the assumed exponential distributions (*m*_1_ = 1 and *m*_2_ = 1.5). Thus, the shape and scale parameters in Weibull distributions had the relationship of *scale* = *m* (log(2))^−1/*shape*^, where *m* was the median. When *shape* = 1, the Weibull distribution is reduced to the assumed exponential distribution. In contrast, the Weibull distribution deviates from the exponential distribution if *shape* ≠ 1 (Supplementary Figure s1). Table 4 listed power and the expected numbers of events under a low (*p* = 0.2) or high switching probability (*p* = 0.6). The shape of 0.5 and 0.75 led to power reductions (< 0.8), while power was improved when shape = 1.25 (> 0.8). To be noted, power was altered in a similar magnitude in both low and high switching probabilities. When the shape changed from 1 to 0.75, for example, power was reduced from 0.8 to 0.53 at a switching probability of 0.2, and from 0.8 to 0.54 at a switching probability of 0.6. In summary, there might be power reductions when the true distributions deviate from the assumed distribution, but the treatment switching seems not to cause additional power reductions. That is, there is no interaction effect between switching probabilities and distribution deviation.

**Table 4:** Power estimation when the true distributions deviate from the assumed distributions

shape		no switching	switching s.dist = “beta”	switching s.dist = “gamma”	switching s.dist = “unif”	switching s.dist = “indepExp”
			Switching probability ***p =*** 0.2			
	*n*	130	166	165	168	169
1.0	Power	0.80	0.80	0.80	0.80	0.80
	E1	104.0	130.8	130.1	132.5	132.8
	E2	89.2	113.8	113.2	115.1	115.8
0.5	Power	0.24	0.26	0.27	0.25	0.25
	E1	96.2	108.7	108.1	110.1	110.3
	E2	77.0	98.4	97.5	99.4	99.9
0.75	Power	0.53	0.53	0.54	0.53	0.53
	E1	96.1	121.0	120.2	122.4	122.6
	E2	83.3	106.2	105.5	107.5	108.1
1.25	Power	0.95	0.94	0.95	0.95	0.95
	E1	109.5	137.9	140.0	139.5	139.7
	E2	94.4	120.6	119.7	122.0	122.7
			Switching probability *p* = 0.6			
	*n*	130	294	301	297	344
1.0	Power	0.80	0.80	0.80	0.80	0.80
	E1	104.0	225.0	230.0	227.1	259.4
	E2	89.2	201.7	206.4	203.9	236.0
0.5	Power	0.24	0.26	0.25	0.26	0.27
	E1	96.2	188.1	192.2	190.2	218.0
	E2	77.0	173.8	178.2	175.8	203.5
0.75	Power	0.53	0.54	0.52	0.53	0.55
	E1	96.1	208.0	212.7	210.3	240.4
	E2	83.3	188.2	192.8	190.2	220.1
1.25	Power	0.95	0.95	0.94	0.95	0.94
	E1	109.5	237.3	242.8	239.8	274.4
	E2	94.4	213.4	218.6	215.6	249.7

E1 and E2: the expected numbers of events in the control group and the experimental group

## Discussion

Our simulation study showed a standard ITT analysis failing to consider treatment switching resulted in a significant reduction of statistical powers especially when the switching probability is high and the switching time is early, which indicated that a much larger sample size was required to maintain the expected power. The sample sizes required in RCTs absence of treatment switching vary from around four-fifths to one-seventh of the sample sizes required in RCTs allowing treatment switching as the switching probability increases. The growth of the relative effectiveness and the switching probability increased the ratio of samples and decreased the power, while the later switching time decreased the ratio of sample sizes and increased the power. Moreover, the event rate in the control group was below the expected event rate (i.e., 1 – *censor.rate*), and decreased with the increased switching probability in trials with treatment switching. When *m*_2_/*m*_1_ = 1.5 and *censor.rate* = 0.2, for example, the event rate E1/ *n* = 0.80 (104/130) under no switching, where E1 was the expected number of events in the control group (Table 2). In contrast, E1/ *n* = 130.8/166 = 0.79 if *p* = 0.2, E1/ *n* = 225/294 = 0.77 if *p* = 0.6, and E1/ *n* = 464.9/627 = 0.74 if *p* = 1.0 when *s.dist* = “beta” (Table 2).

*PowerSwitchingTrial* assumed the effects of the experimental treatment were the same for the participants switched from the control group to the experimental group and those initially assigned to the experimental group, which is the same as the “common treatment effect” assumption made by RPSFTM [8]. In some cases, the assumption may be problematic. The participants switched from the control group to the experimental group may be associated with worse survival. Properly adjusting the accelerated factor *A* may be useful to fit the scenario. Multiplying a constant that is less than 1 to *A* may be a solution, but it is difficult to determine the constant value before clinical trials even if we can borrow the information from previous similar studies.

The assumption of exponential distributions for survival time also limits the flexibility and practice of *PowerSwitchingTrial*. Survival times do not always follow exponential distributions well. Weibull or gamma distribution may be alternatives to exponential distributions. However, it is not easy for clinicians to determine a non-exponential distribution with at least two parameters. Median survival or mean survival is still commonly used, which leads to favoring exponential distributions.

The current version of *PowerSwitchingTrial* is built for two-arm RCTs. It can be extended to multi-arm RCTs, straightforwardly but not simply. More parameters need to be determined in multi-arm clinical trial designs. We plan to extend *PowerSwitchingTrial* for multi-arm RCTs in the near future.

## Conclusions

In this study, we proposed a simulation-based approach, *PowerSwitchingTrial*, to evaluate statistical powers and sample sizes required in RCTs allowing switching in ITT analysis when the logrank test is used. The approach not only provides a preview for power declining but also calculates the required sample size to achieve an expected power when treatment switching occurs. It will provide researchers and clinicians with useful information before RCTs are conducted.

## Supplementary Information

**Additional file 1.**

## Data Availability

Additional file 1: Supplementary Material.

## Abbreviations

ITT: Intention-to-treat
RCT: Randomized controlled trials
RPSFT: Rank preserving structural accelerated failure time models
