# Supplementary material for: Power and sample sizes estimation in clinical trials with treatment switching in intention-to-treat analysis: a simulation study
Source: BMC Med Res Methodol. 2023 Feb 23;23:49. doi: 10.1186/s12874-023-01864-1 (PMC9948351; doi:10.1186/s12874-023-01864-1)
Supplement: Supplementary file 1 — Additional file 1. [file 12874_2023_1864_MOESM1_ESM.docx]

**Supplemental Online Content**

**Power and sample sizes estimation in clinical trials with treatment switching in intention-to-treat analysis: a simulation study**

Lejun Deng^1#^, Chih-Yuan Hsu^2,3#^, Yu Shyr^2,3*^

^1^Montgomery Bell Academy, Nashville, TN, 37205, USA

^2^Department of Biostatistics, Vanderbilt University Medical Center, Nashville, TN 37232, USA

^3^Center for Quantitative Sciences, Vanderbilt University Medical Center, Nashville, TN 37232, USA

*corresponding authors: Yu Shyr (yu.shyr@vanderbilt.edu)

# These authors contributed equally.

- **Details for censoring distribution**
- **Parameters determination in beta, gamma, uniform, and independent exponential distributions via *pt* and *rho***
- **Figure s1:** Weibull survival functions with different shapes and $m_{1}$ = 1 and $m_{2}$ = 1.5.
- **Table s1:** Simulation results for powers and sample sizes under $s = 0.5$ and administrative censoring only (*censor.rate* = “AC.only”).
- **Table s2:** Simulation results for powers and sample sizes under $s = 1$ and administrative censoring only (*censor.rate* = “AC.only”).

**Details for censoring distribution**

The censoring consists of both dropout censoring and administrative censoring. The distribution of the censoring can be expressed as follows:

$$f\left( c | v \right)=d\left( c \right)I\left( 0<c<T_{e}-v \right)+ \bar{D}\left( T_{e}-v \right)I\left( c=T_{e}-v \right),$$

where $v$ is the entry time which follows a uniform distribution $U(0, T_{a})$, and $d\left( c \right)$ and $\bar{D}\left( c \right)$ are the density function and survival function of dropout censoring, respectively. $I(\cdot)$ is the indicator function. For simplicity, we assume a uniform distribution for the dropout censoring, i.e., $d(c) = h^{-1} I(0 < c < h)$. $h$ is determined by the formula of $P\left( C_{1}<T_{1} \right)$ with a given censoring rate of the control group assuming no treatment switching, where $P\left( C_{1}<T_{1} \right)$ is a function of $h$ and can be explicitly expressed as follows:

$$P\left( C_{1}<T_{1} \right)=\int_{0}^{T_{a}} P\left( C_{1}<T_{1} \right|v)f\left( v \right)dv,$$

Where

$$P\left( C_{1}<T_{1} \right|v)=\int_{0}^{T_{e}-v} \int_{c}^{\infty} f\left( c | v \right)f_{T_{1}}\left( t \right)dt dc+ \int_{T_{e}-v}^{\infty} \int_{c}^{\infty} f\left( c | v \right)f_{T_{1}}\left( t \right)dt dc$$

$$=\int_{0}^{T_{e}-v} \int_{c}^{\infty} d\left( c \right)f_{T_{1}}\left( t \right)dt dc+\bar{D}\left( T_{e}-v \right)\int_{T_{e}-v}^{\infty} f_{T_{1}}\left( t \right)dt$$

$$=\int_{0}^{{min(T}_{e}-v, h)} h^{-1}\int_{c}^{\infty} f_{T_{1}}\left( t \right)dt dc+I\left( h>T_{e}-v \right)\left( 1-h^{-1}\left( T_{e}-v \right) \right)\int_{T_{e}-v}^{\infty} f_{T_{1}}\left( t \right)dt$$

$$=\left( h\lambda_{1} \right)^{-1}\left( 1-e^{-\lambda_{1} \min\left( T_{e}-v, h \right)} \right)+I\left( h>T_{e}-v \right)\left( 1-h^{-1}\left( T_{e}-v \right) \right)e^{-\lambda_{1}\left( T_{e}-v \right)}.$$

$$\left( \mathrm{if} T_{1} follows an exponential distribution with the median of m_{1}. \lambda_{1}=\log\left( 2 \right)/m_{1} \right)$$

For $T_{e}-T_{a}<h<T_{e}$,

$P\left( C_{1}<T_{1} \right)=\int_{0}^{T_{e}-h} \left( h\lambda_{1} \right)^{-1}\left( 1-e^{-\lambda_{1} h} \right)f_{V}\left( v \right)dv+\int_{T_{e}-h}^{T_{a}} \left( h\lambda_{1} \right)^{-1}\left( 1-e^{-\lambda_{1} {(T}_{e}-v)} \right)f_{V}\left( v \right)dv$

$$+\int_{T_{e}-h}^{T_{a}} \left( 1-h^{-1}\left( T_{e}-v \right) \right)e^{-\lambda_{1}\left( T_{e}-v \right)}f_{V}\left( v \right)dv$$

$$=\left( h\lambda_{1}T_{a} \right)^{-1}\left( 1-e^{-\lambda_{1} h} \right)\left( T_{e}-h \right)+\left( h\lambda_{1}T_{a} \right)^{-1}\left( T_{a}-\left( T_{e}-h \right) \right)-\left( h\lambda_{1}T_{a} \right)^{-1}\int_{T_{e}-h}^{T_{a}} e^{-\lambda_{1} {(T}_{e}-v)}dv$$

$$+ T_{a}^{-1}\int_{T_{e}-h}^{T_{a}} e^{-\lambda_{1} {(T}_{e}-v)}dv-\left( hT_{a} \right)^{-1}\int_{T_{e}-h}^{T_{a}} \left( T_{e}-v \right)e^{-\lambda_{1} {(T}_{e}-v)}dv$$

$$=\left( h\lambda_{1}T_{a} \right)^{-1}\left\{ \left( 1-e^{-\lambda_{1} h} \right)\left( T_{e}-h \right)+\left( T_{a}-\left( T_{e}-h \right) \right)-{\lambda_{1}^{-1}b}_{2}\left( h \right)+hb_{2}\left( h \right)-(b_{1}\left( h \right)+{\lambda_{1}^{-1}b}_{2}\left( h \right)) \right\},$$

where $b_{1}\left( x \right)=\left( T_{e}-T_{a} \right)e^{-\lambda_{1} {(T}_{e}-T_{a})}-x e^{-\lambda_{1}x}$ and $b_{2}\left( x \right)=e^{-\lambda_{1} {(T}_{e}-T_{a})}-e^{-\lambda_{1}x}$.

For $h\leq T_{e}-T_{a}$,

$P\left( C_{1}<T_{1} \right)=\int_{0}^{T_{a}} \left( h\lambda_{1} \right)^{-1}\left( 1-e^{-\lambda_{1} h} \right)f_{V}\left( v \right)dv=\left( h\lambda_{1} \right)^{-1}\left( 1-e^{-\lambda_{1} h} \right)$.

For $T_{e}\leq h$,

$$P\left( C_{1}<T_{1} \right)=\int_{0}^{T_{a}} \left( h\lambda_{1} \right)^{-1}\left( 1-e^{-\lambda_{1} \left( T_{e}-v \right)} \right)f_{V}\left( v \right)dv+\int_{0}^{T_{a}} \left( 1-h^{-1}\left( T_{e}-v \right) \right)e^{-\lambda_{1}\left( T_{e}-v \right)}f_{V}\left( v \right)dv$$

$$=\left( h\lambda_{1}T_{a} \right)^{-1}\left( T_{a}-\lambda_{1}^{-1}b_{2}\left( T_{e} \right)+hb_{2}\left( T_{e} \right)-(b_{1}\left( T_{e} \right)+{\lambda_{1}^{-1}b}_{2}\left( T_{e} \right)) \right).$$

$$\left( =\left( h\lambda_{1} \right)^{-1}\left( 1-e^{-\lambda_{1}T_{e}}+h\lambda_{1}e^{-\lambda_{1}T_{e}}-\lambda_{1}T_{e}e^{-\lambda_{1}T_{e}} \right) if T_{a}=0 \right)$$

**Parameters determination in beta, gamma, uniform, and independent exponential distributions via *pt* and *rho***

We consider using $pt$ and *rho* to determine the parameters in the assumed distributions for the switching time, where $pt=E(s)/E\left( T_{1} \right)$ denotes the ratio of the average switching time to the average survival time of the control group, and *rho* denotes the correlation between $s=XT_{1}$ and $T_{1}$. When $pt$ and $rho$ are given, the parameters in the assumed distributions can be obtained through solving the two equations: $pt=E(s)/E\left( T_{1} \right)=E(X)$ and

$$rho=cor\left( s, T_{1} \right)=\frac{E\left( XT_{1}^{2} \right)-E\left( XT_{1} \right)E\left( T_{1} \right)}{\sqrt{Var\left( s \right)}\sqrt{Var\left( T_{1} \right)}}=\frac{E\left( X \right)\sqrt{Var\left( T_{1} \right)}}{\sqrt{E\left( X^{2} \right)Var\left( T_{1} \right)+Var\left( X \right)\left( E\left( T_{1} \right) \right)^{2}}}$$

$$=\frac{E(X)}{\sqrt{E\left( X^{2} \right)+Var(X)}} \left( \mathrm{if} T_{1} follows an exponential distribution \right),$$

where

$$Var\left( s \right)=E\left( X^{2} \right)E\left( T_{1}^{2} \right)-\left( E\left( X \right) \right)^{2}\left( E\left( T_{1} \right) \right)^{2}$$

$$=E\left( X^{2} \right)E\left( T_{1}^{2} \right)-E\left( X^{2} \right)\left( E\left( T_{1} \right) \right)^{2}+{E\left( X^{2} \right)\left( E\left( T_{1} \right) \right)^{2}-\left( E\left( X \right) \right)}^{2}\left( E\left( T_{1} \right) \right)^{2}$$

$$=E\left( X^{2} \right)Var\left( T_{1} \right)+Var\left( X \right)\left( E\left( T_{1} \right) \right)^{2}.$$

When $s=XT_{1}$ and $X\sim beta(a,b)$, $pt=a/(a+b)$ and $rho=\left\{ \frac{a}{a+b} \right\}/ \left\{ \left( \frac{a}{a+b} \right)^{2}+\frac{2ab}{\left( a+b \right)^{2}\left( a+b+1 \right)} \right\}^{1/2}$. When $s=XT_{1}$ and $X\sim gamma(a,b)$, $pt=a/b$ and $rho=\left\{ \frac{a}{b} \right\}/ \left\{ \left( \frac{a}{b} \right)^{2}+\frac{2a}{b^{2}} \right\}^{1/2}$. When $s$ follows an exponential distribution and is independent of $T_{1}$, the mean parameter can be determined by $E\left( s \right)=pt E\left( T_{1} \right)=pt {\times m}_{1}/log(2).$ When $s=XT_{1}$ and $X$ follows a uniform distribution $U(0, 1)$, i.e., $s$ follows a uniform distribution $U\left( 0, T_{1} \right)$, then $pt$ equals 0.5 and $rho$ is a constant (= 0.775 if $T_{1}$ follows an exponential distribution).

**
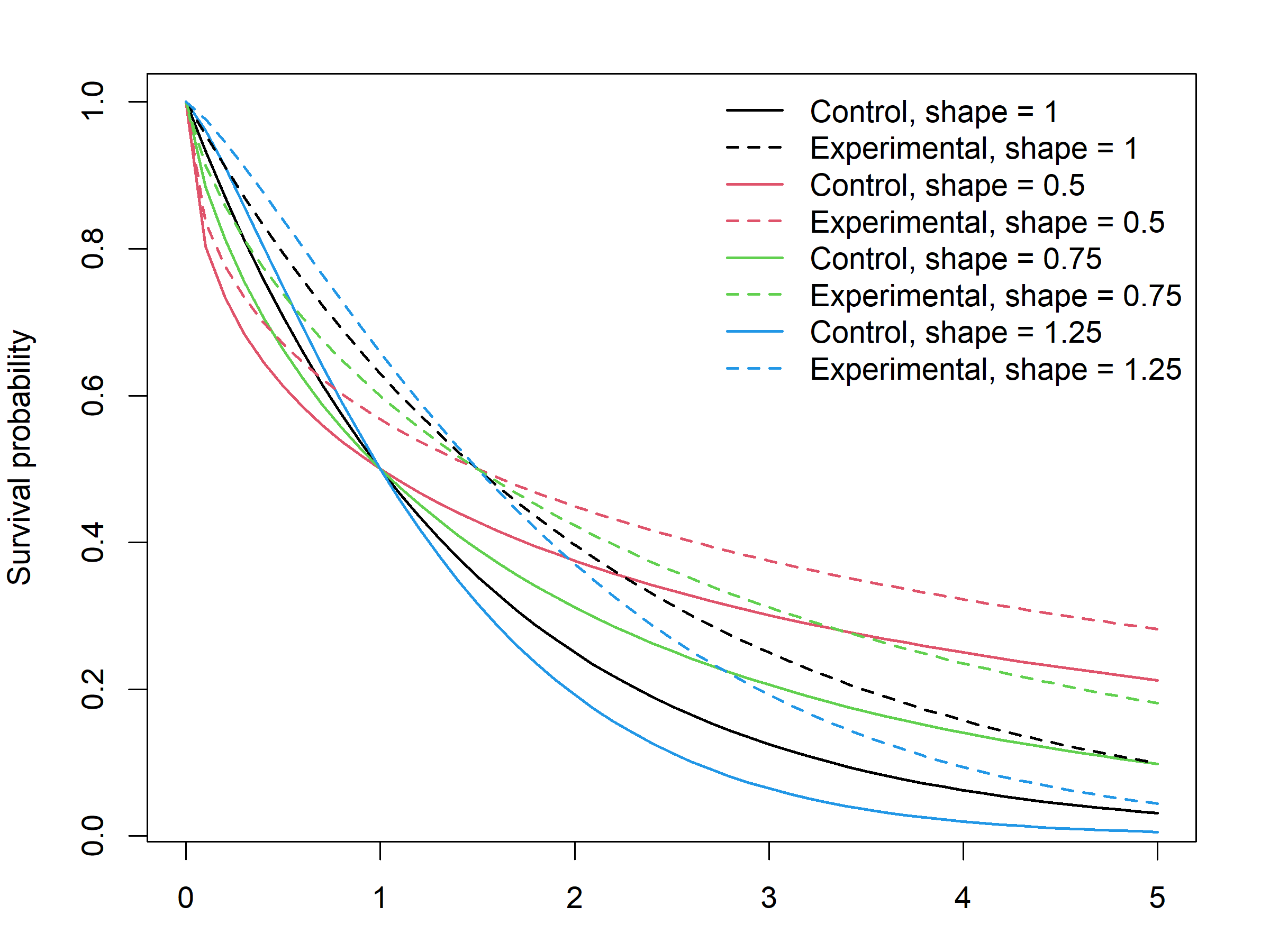
**

**Figure s1.** Weibull survival functions with different shapes and $m_{1}$ = 1 and $m_{2}$ = 1.5.

**Table s1.** Simulation results for powers and sample sizes under $s = 0.5$ and administrative censoring only (*censor.rate* = “AC.only”). *power* = 0.8, *alpha* = 0.05, $m_{1}$ = 1, $T_{a}$ = 3, $T_{e}$ = 5.

| $\boldsymbol{r}$ | $\boldsymbol{p}$ | $\boldsymbol{m}_{\boldsymbol{2}}\boldsymbol{/}\boldsymbol{m}_{\boldsymbol{1}}$ | $\boldsymbol{n}_{\boldsymbol{no-s}}$  (no switch) | $\boldsymbol{n}_{\boldsymbol{S}}$  (switch) | Ratio of  $\boldsymbol{n}_{\boldsymbol{s}}\boldsymbol{/}\boldsymbol{n}_{\boldsymbol{no-s}}$ | Power based on $\boldsymbol{n}_{\boldsymbol{no-s}}$ |
| --- | --- | --- | --- | --- | --- | --- |
| 1 | 0.2 | 1.25 | 369 | 501 | 1.358 | 0.668 |
|  |  | 1.50 | 115 | 160 | 1.391 | 0.665 |
|  |  | 1.75 | 63 | 87 | 1.396 | 0.652 |
|  |  | 2.00 | 42 | 59 | 1.404 | 0.647 |
| 1 | 0.4 | 1.25 | 369 | 715 | 1.938 | 0.525 |
|  |  | 1.50 | 115 | 234 | 2.035 | 0.516 |
|  |  | 1.75 | 63 | 129 | 2.048 | 0.505 |
|  |  | 2.00 | 42 | 87 | 2.071 | 0.504 |
| 1 | 0.6 | 1.25 | 369 | 1064 | 2.883 | 0.365 |
|  |  | 1.50 | 115 | 364 | 3.165 | 0.355 |
|  |  | 1.75 | 63 | 199 | 3.159 | 0.353 |
|  |  | 2.00 | 42 | 139 | 3.310 | 0.350 |
| 1 | 0.8 | 1.25 | 369 | 1849 | 5.011 | 0.246 |
|  |  | 1.50 | 115 | 616 | 5.617 | 0.223 |
|  |  | 1.75 | 63 | 343 | 5.444 | 0.227 |
|  |  | 2.00 | 42 | 233 | 5.548 | 0.235 |
| 1 | 1.0 | 1.25 | 369 | 3728 | 10.103 | 0.142 |
|  |  | 1.50 | 115 | 1230 | 10.696 | 0.141 |
|  |  | 1.75 | 63 | 691 | 10.968 | 0.138 |
|  |  | 2.00 | 42 | 487 | 11.595 | 0.134 |
| 2 | 0.2 | 1.25 | 271 | 371 | 1.369 | 0.675 |
|  |  | 1.50 | 82 | 117 | 1.427 | 0.652 |
|  |  | 1.75 | 45 | 66 | 1.467 | 0.663 |
|  |  | 2.00 | 31 | 46 | 1.484 | 0.665 |
| 2 | 0.4 | 1.25 | 271 | 535 | 1.974 | 0.526 |
|  |  | 1.50 | 82 | 170 | 2.073 | 0.507 |
|  |  | 1.75 | 45 | 94 | 2.089 | 0.515 |
|  |  | 2.00 | 31 | 63 | 2.032 | 0.501 |
| 2 | 0.6 | 1.25 | 271 | 810 | 2.980 | 0.375 |
|  |  | 1.50 | 82 | 262 | 3.195 | 0.362 |
|  |  | 1.75 | 45 | 148 | 3.289 | 0.361 |
|  |  | 2.00 | 31 | 102 | 3.290 | 0.366 |
| 2 | 0.8 | 1.25 | 271 | 1395 | 5.148 | 0.239 |
|  |  | 1.50 | 82 | 457 | 5.573 | 0.233 |
|  |  | 1.75 | 45 | 252 | 5.600 | 0.232 |
|  |  | 2.00 | 31 | 178 | 5.742 | 0.238 |
| 2 | 1.0 | 1.25 | 271 | 2796 | 10.314 | 0.147 |
|  |  | 1.50 | 82 | 937 | 11.427 | 0.139 |
|  |  | 1.75 | 45 | 513 | 11.400 | 0.148 |
|  |  | 2.00 | 31 | 361 | 11.645 | 0.161 |

$n_{s}$ and $n_{no-s}$ denote the required sample size with and without treatment switching, respectively.

**Table s2.** Simulation results for powers and sample sizes under $s = 1$ and administrative censoring only (*censor.rate* = “AC.only”). *power* = 0.8, *alpha* = 0.05, $m_{1}$ = 1, $T_{a}$ = 3, $T_{e}$ = 5.

| $\boldsymbol{r}$ | $\boldsymbol{p}$ | $\boldsymbol{m}_{\boldsymbol{2}}\boldsymbol{/}\boldsymbol{m}_{\boldsymbol{1}}$ | $\boldsymbol{n}_{\boldsymbol{no-s}}$  (no switch) | $\boldsymbol{n}_{\boldsymbol{S}}$  (switch) | Ratio of  $\boldsymbol{n}_{\boldsymbol{s}}\boldsymbol{/}\boldsymbol{n}_{\boldsymbol{no-s}}$ | Power based on $\boldsymbol{n}_{\boldsymbol{no-s}}$ |
| --- | --- | --- | --- | --- | --- | --- |
| 1 | 0.2 | 1.25 | 369 | 446 | 1.209 | 0.716 |
|  |  | 1.50 | 115 | 143 | 1.243 | 0.700 |
|  |  | 1.75 | 63 | 81 | 1.286 | 0.693 |
|  |  | 2.00 | 42 | 56 | 1.310 | 0.689 |
| 1 | 0.4 | 1.25 | 369 | 560 | 1.518 | 0.610 |
|  |  | 1.50 | 115 | 179 | 1.557 | 0.620 |
|  |  | 1.75 | 63 | 98 | 1.556 | 0.595 |
|  |  | 2.00 | 42 | 68 | 1.619 | 0.600 |
| 1 | 0.6 | 1.25 | 369 | 706 | 1.913 | 0.507 |
|  |  | 1.50 | 115 | 231 | 2.009 | 0.504 |
|  |  | 1.75 | 63 | 128 | 2.032 | 0.513 |
|  |  | 2.00 | 42 | 86 | 2.048 | 0.514 |
| 1 | 0.8 | 1.25 | 369 | 916 | 2.482 | 0.423 |
|  |  | 1.50 | 115 | 299 | 2.600 | 0.409 |
|  |  | 1.75 | 63 | 168 | 2.667 | 0.418 |
|  |  | 2.00 | 42 | 119 | 2.833 | 0.407 |
| 1 | 1.0 | 1.25 | 369 | 1278 | 3.463 | 0.324 |
|  |  | 1.50 | 115 | 401 | 3.487 | 0.313 |
|  |  | 1.75 | 63 | 225 | 3.514 | 0.313 |
|  |  | 2.00 | 42 | 156 | 3.714 | 0.300 |
| 2 | 0.2 | 1.25 | 271 | 329 | 1.214 | 0.723 |
|  |  | 1.50 | 82 | 103 | 1.256 | 0.716 |
|  |  | 1.75 | 45 | 57 | 1.267 | 0.711 |
|  |  | 2.00 | 31 | 41 | 1.323 | 0.708 |
| 2 | 0.4 | 1.25 | 271 | 409 | 1.509 | 0.634 |
|  |  | 1.50 | 82 | 131 | 1.598 | 0.628 |
|  |  | 1.75 | 45 | 72 | 1.600 | 0.621 |
|  |  | 2.00 | 31 | 48 | 1.548 | 0.613 |
| 2 | 0.6 | 1.25 | 271 | 516 | 1.904 | 0.528 |
|  |  | 1.50 | 82 | 168 | 2.049 | 0.521 |
|  |  | 1.75 | 45 | 92 | 2.044 | 0.495 |
|  |  | 2.00 | 31 | 64 | 2.065 | 0.503 |
| 2 | 0.8 | 1.25 | 271 | 684 | 2.524 | 0.434 |
|  |  | 1.50 | 82 | 223 | 2.720 | 0.418 |
|  |  | 1.75 | 45 | 124 | 2.756 | 0.421 |
|  |  | 2.00 | 31 | 83 | 2.677 | 0.429 |
| 2 | 1.0 | 1.25 | 271 | 918 | 3.387 | 0.329 |
|  |  | 1.50 | 82 | 306 | 3.732 | 0.312 |
|  |  | 1.75 | 45 | 168 | 3.733 | 0.340 |
|  |  | 2.00 | 31 | 116 | 3.742 | 0.329 |

$n_{s}$ and $n_{no-s}$ denote the required sample size with and without treatment switching, respectively.
